# Supplementary material for: Metabolomics identifies and validates serum androstenedione as novel biomarker for diagnosing primary angle closure glaucoma and predicting the visual field progression
Source: eLife. 2024 Feb 15;12:RP91407. doi: 10.7554/eLife.91407 (PMC10942597; doi:10.7554/eLife.91407)
Supplement: Supplementary file 1. [file elife-91407-supp1.docx]

**Supplementary file 1**

| Score OPLS-DA Plot | R(MetaboAnalystR) version 1.0.1 |
| --- | --- |
| Volcano plots | R(ggplot2), version 3.3.0 |
| Venn diagram | R(VennDiagram) version 1.6.20 |
| ComplexHeatmap | R(ComplexHeatmap) version 2.6.2 |
| 2-column heatmap | R(pheatmap) Version 1.0.12 |
| scatter diagram ofcomponents | R(ggplot2) Version 3.3.6 |
| violin plot | R(ggplot2) version 3.3.6 |
| KEGG | R(ggplot2) version 3.3.0 |
| ROC curve | R(ggplot2) Version 1.18.0 |
| Spearman analysis | GraphPad Prism 9 |
| Random Forest | randomForest (Version 4.6-14) |
| support vector machine-rbf | kernlab (Version 0.9-29) |
| support vector machine-poly | kernlab (Version 0.9-29) |
| Logistic Regression | glmnet (Version 4.1-3) |
| Nearest Neighbor | kknn (Version 1.3.1) |
| GaussianNB | naivebayes (Version 0.9.7) |
| Binding mode of molecular docking | AutodockVina 1.2.2 |

**List of statistical approach and packages**
