# Supplementary material for: Metabolomics identifies and validates serum androstenedione as novel biomarker for diagnosing primary angle closure glaucoma and predicting the visual field progression
Source: eLife. 2024 Feb 15;12:RP91407. doi: 10.7554/eLife.91407 (PMC10942597; doi:10.7554/eLife.91407)
Supplement: Supplementary file 2. [file elife-91407-supp2.docx]

**Supplementary file 2**

|  | Cataract | PACG | t/Fisher | p |
| --- | --- | --- | --- | --- |
| Number (n) | 11 | 7 |  |  |
| Age (Years) | 62.55±6.23 | 60.00±4.00 | 0.96 | 0.35 |
| Sex（Male，%） | 3 (27.3) | 3 (42.9) | 0.47 | 0.63 |
| BMI (Kg/m^2^) | 23.99±3.29 | 22.18±3.38 | 1.01 | 0.33 |
| Hypercholesterolemia (Yes，%) | 1 (9.1) | 1 (14.3) | 0.12 | 1 |
| Hypertension (Yes，%) | 3 (27.3) | 2 (28.6) | 0.00 | 1 |
| Diabetes (Yes，%) | 2 (18.2) | 1 (14.3) | 0.05 | 1 |
| Smoking (Yes，%) | 1 (9.1) | 1 (14.3) | 0.12 | 1 |
| Drinking (Yes，%) | 2 (18.2) | 1 (14.3) | 0.05 | 1 |
| Duration (Months) |  | 4.43±1.81 |  |  |
| VCDR |  | 0.71±0.23 |  |  |
| AL (mm) |  | 22.30±1.19 |  |  |
| ACD (mm) |  | 1.81±0.11 |  |  |
| CCT (um) |  | 549.85±62.88 |  |  |
| MS (dB) |  | 12.08±10.07 |  |  |
| MD (dB) |  | 15.53±10.47 |  |  |

**The clinical and demographic characteristics of PACG and cataract subjects in the supplemental phase**
