# Supplementary material for: Metabolomics identifies and validates serum androstenedione as novel biomarker for diagnosing primary angle closure glaucoma and predicting the visual field progression
Source: eLife. 2024 Feb 15;12:RP91407. doi: 10.7554/eLife.91407 (PMC10942597; doi:10.7554/eLife.91407)
Supplement: Supplementary file 3. [file elife-91407-supp3.docx]

**Supplementary file 3**

|  | Pretreatment | Posttreatment | t/Fisher | p |
| --- | --- | --- | --- | --- |
| Number (n) | 9 | 9 |  |  |
| Age (Years) | 65.25±11.31 |  |  |  |
| Sex（Male，%） | 3 (33.3) |  |  |  |
| BMI (Kg/m^2^) | 24.64±3.36 |  |  |  |
| Hypercholesterolemia (Yes，%) | 0 (0) |  |  |  |
| Hypertension (Yes，%) | 2 (22.2) |  |  |  |
| Diabetes (Yes，%) | 2 (22.2) |  |  |  |
| Smoking (Yes，%) | 1 (11.1) |  |  |  |
| Drinking (Yes，%) | 2 (22.2) |  |  |  |
| Duration (Months) | 6.00±4.47 | 9.00±4.47 | 1.42 | 0.17 |
| VCDR | 0.54±0.11 | 0.56±0.18 | 0.28 | 0.78 |
| AL (mm) | 22.70±1.06 | 22.54±1.04 | 0.32 | 0.75 |
| ACD (mm) | 1.77±0.30 | 1.80±0.32 | 0.21 | 0.84 |
| CCT (um) | 547.28±42.16 | 556.21±38.46 | 0.47 | 0.65 |
| MS (dB) | 15.21±6.46 | 14.93±6.80 | 0.09 | 0.93 |
| MD (dB) | 12.50±8.86 | 13.15±9.31 | 0.15 | 0.88 |

**The clinical and demographic characteristics of same PACG patients between pretreatment and posttreatment**
