# Supplementary material for: Metabolomics identifies and validates serum androstenedione as novel biomarker for diagnosing primary angle closure glaucoma and predicting the visual field progression
Source: eLife. 2024 Feb 15;12:RP91407. doi: 10.7554/eLife.91407 (PMC10942597; doi:10.7554/eLife.91407)
Supplement: Supplementary file 4. [file elife-91407-supp4.docx]

**Supplementary file 4**

| Compounds | Class | T-test_P | FC_Mean | FDR |
| --- | --- | --- | --- | --- |
| DHA | FA | 0.021 | 0.79 | 0.05 |
| Vanillylmandelic acid | Benzene and substituted derivatives | 0.0086 | 0.86 | 0.06 |
| FFA(18:4) | FA | 0.017 | 0.75 | 0.06 |
| FFA(22:6) | FA | 0.021 | 0.79 | 0.05 |
| Leu-Ile | Amino acid and Its metabolomics | 0.062 | 1.20 | 0.32 |
| Theobromine | Nucleotide And Its metabolomics | 0.051 | 2.27 | 0.93 |
| 5-Aminolevulinate | Organic acid And Its derivatives | 0.0098 | 1.34 | 0.04 |
| 2-Pyrrolidinone | Heterocyclic compounds | 0.013 | 0.79 | 0.07 |
| 2-Mercaptobenzothiazole | Heterocyclic compounds | 0.074 | 1.25 | 0.06 |
| Androstenedione | Hormones and hormone related compunds | 0.015 | 1.21 | 0.26 |
| Guanidine | Alcohol and amines | 0.060 | 0.87 | 0.27 |
| Ser-Leu | Amino acid and Its metabolomics | 0.051 | 1.19 | 0.44 |
| C11H18N2O4 | Amino acid and Its metabolomics | 0.048 | 0.73 | 0.04 |
| Cyclo(Pro-Leu) | Amino acid and Its metabolomics | 0.022 | 0.71 | 0.10 |
| C16H10O6 | Organic acid And Its derivatives | <0.001 | 1.19 | 0.00 |
| Atenolol | others | 0.017 | 0.85 | 0.08 |
| 16,16-dimethyl-PGA1 | others | 0.00021 | 0.72 | 0.01 |
| C19H39O7P | others | 0.013 | 0.81 | 0.04 |
| C12H16O5 | Alcohol and amines | 0.081 | 0.77 | 0.21 |
| 6-Ketomyristic acid | Organic acid And Its derivatives | 0.017 | 0.75 | 0.06 |
| C27H46O3 | Heterocyclic compounds | 0.014 | 0.76 | 0.23 |
| 伪-Cyperone | Heterocyclic compounds | 0.0064 | 1.41 | 0.27 |
| 3-Hydroxycapric acid | Organic acid And Its derivatives | 0.015 | 0.76 | 0.05 |
| Linalyl cinnamate | Organic acid And Its derivatives | 0.070 | 0.86 | 0.16 |
| C16H17N3OS | Heterocyclic compounds | 0.0030 | 1.50 | 0.15 |
| Cadiamine | Alcohol and amines | <0.001 | 0.71 | 0.00 |
| C10H14O4 | Organic acid And Its derivatives | 0.045 | 0.75 | 0.19 |
| Dihydro Isorescinnamine | Organic acid And Its derivatives | 0.067 | 0.76 | 0.17 |
| Phe His Glu | Amino acid and Its metabolomics | 0.044 | 1.22 | 0.31 |
| Ser Ala Lys Lys | Amino acid and Its metabolomics | <0.001 | 0.79 | 0.01 |
| Thr Asn Phe Asp | Amino acid and Its metabolomics | <0.001 | 1.78 | 0.01 |
| Brassinolide | Organic acid And Its derivatives | 0.0089 | 0.70 | 0.03 |

**The differential metabolites associated with PACG and their fold-changes in discovery set 1**
