# Supplementary material for: Metabolomics identifies and validates serum androstenedione as novel biomarker for diagnosing primary angle closure glaucoma and predicting the visual field progression
Source: eLife. 2024 Feb 15;12:RP91407. doi: 10.7554/eLife.91407 (PMC10942597; doi:10.7554/eLife.91407)
Supplement: Supplementary file 5. [file elife-91407-supp5.docx]

**Supplementary file 5**

| Compounds | Class | T-test_P | FC_Mean | FDR |
| --- | --- | --- | --- | --- |
| DHA | FA | 0.080 | 0.87 | 0.25 |
| Vanillylmandelic acid | Benzene and substituted derivatives | 0.019 | 0.83 | 0.11 |
| FFA(18:4) | FA | 0.078 | 0.79 | 0.48 |
| FFA(22:6) | FA | 0.080 | 0.87 | 0.25 |
| Leu-Ile | Amino acid and Its metabolomics | 0.027 | 1.30 | 0.27 |
| Theobromine | Nucleotide And Its metabolomics | 0.086 | 0.59 | 0.56 |
| 5-Aminolevulinate | Organic acid And Its derivatives | 0.013 | 0.79 | 0.37 |
| 2-Pyrrolidinone | Heterocyclic compounds | <0.001 | 1.47 | 0.00 |
| 2-Mercaptobenzothiazole | Heterocyclic compounds | <0.001 | 2.61 | 0.00 |
| Androstenedione | Hormones and hormone related compunds | 0.036 | 1.15 | 0.72 |
| Guanidine | Alcohol and amines | 0.013 | 1.17 | 0.11 |
| Ser-Leu | Amino acid and Its metabolomics | 0.0052 | 1.37 | 0.06 |
| C11H18N2O4 | Amino acid and Its metabolomics | 0.075 | 1.16 | 0.48 |
| Cyclo(Pro-Leu) | Amino acid and Its metabolomics | 0.011 | 0.77 | 0.07 |
| C16H10O6 | Organic acid And Its derivatives | 0.020 | 1.20 | 0.38 |
| Atenolol | others | 0.0018 | 1.21 | 0.17 |
| 16,16-dimethyl-PGA1 | others | 0.037 | 1.19 | 0.31 |
| C19H39O7P | others | 0.060 | 0.86 | 0.24 |
| C12H16O5 | Alcohol and amines | 0.057 | 0.76 | 0.11 |
| 6-Ketomyristic acid | Organic acid And Its derivatives | 0.032 | 0.81 | 0.45 |
| C27H46O3 | Heterocyclic compounds | 0.011 | 1.20 | 0.07 |
| 伪-Cyperone | Heterocyclic compounds | <0.001 | 1.84 | 0.00 |
| 3-Hydroxycapric acid | Organic acid And Its derivatives | 0.063 | 0.85 | 0.56 |
| Linalyl cinnamate | Organic acid And Its derivatives | <0.001 | 1.25 | 0.00 |
| C16H17N3OS | Heterocyclic compounds | <0.001 | 1.93 | 0.00 |
| Cadiamine | Alcohol and amines | 0.062 | 1.16 | 0.38 |
| C10H14O4 | Organic acid And Its derivatives | 0.071 | 0.80 | 0.13 |
| Dihydro Isorescinnamine | Organic acid And Its derivatives | 0.026 | 1.50 | 0.23 |
| Phe His Glu | Amino acid and Its metabolomics | 0.0012 | 1.36 | 0.19 |
| Ser Ala Lys Lys | Amino acid and Its metabolomics | 0.0035 | 1.16 | 0.14 |
| Thr Asn Phe Asp | Amino acid and Its metabolomics | <0.001 | 1.75 | 0.00 |
| Brassinolide | Organic acid And Its derivatives | 0.073 | 0.85 | 0.57 |

**The differential metabolites associated with PACG and their fold-changes in discovery set 2**
