# Supplementary material for: Metabolomics identifies and validates serum androstenedione as novel biomarker for diagnosing primary angle closure glaucoma and predicting the visual field progression
Source: eLife. 2024 Feb 15;12:RP91407. doi: 10.7554/eLife.91407 (PMC10942597; doi:10.7554/eLife.91407)
Supplement: Supplementary file 6. [file elife-91407-supp6.docx]

**Supplementary file 6**

| Compounds | Formula | Polarity | m/z | RT(second) | Adduct | m/z Error(ppm) | RT Error(s) | MS/MS Similarity Score(Dot Product) |
| --- | --- | --- | --- | --- | --- | --- | --- | --- |
| DHA | C22H32O2 | negative | 327.2 | 10.92 | [M-H]- | -1.22471E-10 | 0.00 | 0.87 |
| 2-hydroxy-2-(4-hydroxy-3-methoxyphenyl)acetic acid | C9H10O5 | negative | 197 | 6.64 | [M-H]- | -2.66714E-10 | -0.01 | - |
| FFA(18:4) | C18H28O2 | negative | 275.2 | 10.06 | [M-H]- | -3.2298E-11 | 0.14 | - |
| FFA(22:6) | C22H32O2 | negative | 327.2 | 10.92 | [M-H]- | -1.22471E-10 | 0.24 | 0.91 |
| Leu-Ile | C12H24N2O3 | negative | 243 | 2.98 | [M-H]- | -1.35063E-09 | 0.10 | - |
| Theobromine | C7H8N4O2 | positive | 181 | 2.13 | [M+H]+ | -3.59482E-10 | -0.14 | - |
| 5-Aminolevulinate | C5H9NO3 | positive | 132.1 | 0.71 | [M+H]+ | 3.18613E-10 | 0.01 | - |
| 2-Pyrrolidinone | C4H7NO | positive | 86.1 | 1.56 | [M+H]+ | 5.55373E-10 | 0.00 | - |
| 2-Mercaptobenzothiazole | C7H5NS2 | positive | 168 | 5.71 | [M+H]+ | 8.18091E-11 | -0.02 | - |
| Androstenedione | C19H26O2 | positive | 287.2 | 7.14 | [M+H]+ | 2.34806E-11 | -0.02 | 0.90 |
| Guanidine | CH5N3 | positive | 60.1 | 0.7 | [M+H]+ | 8.74758E-10 | 0.00 | - |
| Ser-Leu | C9H18N2O4 | positive | 219.1 | 1.87 | [M+H]+ | -6.45463E-08 | 0.00 | - |
| N(Alpha)-Acetyl-Epsilon-(2-Propenal)Lysine | C11H18N2O4 | positive | 243.1 | 3.3 | [M+H]+ | -1.10108E-10 | 0.01 | - |
| Cyclo(Pro-Leu) | C11H18N2O2 | positive | 211 | 3.68 | [M+H]+ | -1.28406E-09 | -0.13 | - |
| 3,8-Dihydroxy-1-methylanthraquinone-2-carboxylic acid | C16H10O6 | negative | 297 | 6.49 | [M-H]- | -1.60166E-10 | -0.01 | 0.74 |
| Atenolol | C14H22N2O3 | positive | 308.2 | 2.81 | [M+CH3CN+H]+ | 1.38855E-10 | 0.13 | 0.68 |
| 16,16-dimethyl-PGA1 | C22H36O4 | positive | 365.3 | 9.79 | [M+H]+ | 1.06078E-10 | -0.03 | 0.65 |
| 1-Hexadecanoyl-2-sn-glycero-3-phosphate | C19H39O7P | negative | 409.2 | 10.81 | [M-H]- | -1.05645E-10 | 0.06 | 0.71 |
| 2-Furanpropanoic acid, 3-carboxy-4-methyl-5-propyl- | C12H16O5 | negative | 241.1 | 6.01 | [M-H]- | 8.33082E-09 | -0.02 | 0.87 |
| 6-Ketomyristic acid | C14H26O3 | negative | 241.2 | 8.54 | [M-H]- | -8.22822E-07 | -0.01 | 0.63 |
| 7α,24(S)-Dihydroxycholesterol | C27H46O3 | positive | 465.4 | 10.12 | [M+HCOO+2H]+ | 1.32197E-10 | 0.07 | 0.69 |
| α-Cyperone | C15H22O | positive | 219.2 | 5.67 | [M+H]+ | 1.50958E-10 | -0.11 | 0.75 |
| 3-Hydroxycapric acid | C10H20O3 | negative | 187.1 | 6.51 | [M-H]- | -2.19224E-10 | -0.01 | 0.88 |
| Linalyl cinnamate | C19H24O2 | negative | 283.2 | 9.7 | [M-H]- | -8.24489E-07 | 0.02 | 0.61 |
| 6-Hydroxy-5,7-dimethyl-2-methylamino-4-(3-pyridylmethyl)benzothiazole | C16H17N3OS | positive | 341.1 | 5.67 | [M+CH3CN+H]+ | -3.0758E-11 | -0.15 | 0.61 |
| Cadiamine | C15H26N2O3 | positive | 582.4 | 3.41 | [2M+NH4]+ | 2.01988E-11 | 0.13 | 0.53 |
| cis-5,6-Dihydroxy-4-isopropylcyclohexa-1,3-dienecarboxylic acid | C10H14O4 | negative | 181.1 | 6.01 | [M+H-H2O]+ | 5.45714E-11 | -0.06 | 0.60 |
| Dihydro Isorescinnamine | C35H44N2O9 | positive | 654.3 | 11 | [M+NH4]+ | -7.35654E-12 | -0.03 | 0.64 |
| Phe His Glu | C20H25N5O6 | positive | 478.2 | 4.95 | [M+HCOO+2H]+ | 4.52595E-11 | -0.12 | 0.61 |
| Ser Ala Lys Lys | C18H36N6O6 | positive | 433.3 | 3.49 | [M+H]+ | 7.02504E-11 | 0.16 | 0.70 |
| Thr Asn Phe Asp | C21H29N5O9 | negative | 494.2 | 5.07 | [M-H]- | 7.01217E-12 | -0.01 | 0.71 |
| Brassinolide | C28H48O6 | negative | 239.2 | 7.93 | [M-2H]- | 1.14293E-10 | 0.00 | 0.77 |

**The detecting parameters of 32 differential metabolites**
