# Supplementary material for: Metabolomics identifies and validates serum androstenedione as novel biomarker for diagnosing primary angle closure glaucoma and predicting the visual field progression
Source: eLife. 2024 Feb 15;12:RP91407. doi: 10.7554/eLife.91407 (PMC10942597; doi:10.7554/eLife.91407)
Supplement: Supplementary file 7. [file elife-91407-supp7.docx]

**Supplementary file 7**

|  | Type | Discovery phase | | Validation phase 1 | | Validation phase 2 | |
| --- | --- | --- | --- | --- | --- | --- | --- |
|  |  | P value | FDR | P value | FDR | P value | FDR |
| Androstenedione | Logit | 0.034 | 0.019 | 0.043 | 0.078 | 0.001 | 0.015 |
| Androstenedione | Probit | 0.016 | 0.089 | 0.040 | 0.031 | 0.001 | 0.009 |

Adjust for age, sex, BMI, hypercholesterolemia, hypertension, diabetes, smoking, drinking, and duration.

**The relationship between androstenedione and risk of PACG**
