# Supplementary material for: Metabolomics identifies and validates serum androstenedione as novel biomarker for diagnosing primary angle closure glaucoma and predicting the visual field progression
Source: eLife. 2024 Feb 15;12:RP91407. doi: 10.7554/eLife.91407 (PMC10942597; doi:10.7554/eLife.91407)
Supplement: Supplementary file 8. [file elife-91407-supp8.docx]

**Supplementary file 8**

|  | Cutoff value of model score | AUC (95%CI) | Sensitivity | Specificity | Accuracy | NPV | PPV |
| --- | --- | --- | --- | --- | --- | --- | --- |
| PACG vs. NC |  |  |  |  |  |  |  |
| Discovery phase |  |  |  |  |  |  |  |
| Discovery set 1 | 0.50 | 1.00 (1.00-1.00) | 1.00 | 1.00 | 1.00 | 1.00 | 1.00 |
| Discovery set 2 | 0.50 | 0.85 (0.80-0.90) | 0.86 | 0.74 | 0.81 | 0.81 | 0.81 |
| Validation phase 1 | 0.50 | 0.87 (0.80-0.95) | 0.79 | 0.83 | 0.80 | 0.77 | 0.90 |
| Validation phase 2 | 0.50 | 0.86 (0.81-0.91) | 0.74 | 0.97 | 0.85 | 0.75 | 0.97 |
| Mild + Moderate vs. Severe |  |  |  |  |  |  |  |
| Discovery phase |  |  |  |  |  |  |  |
| Discovery set 1 | 0.62 | 0.94 (0.89-0.99) | 0.87 | 0.89 | 0.88 | 0.81 | 0.93 |
| Discovery set 2 | 0.62 | 0.93 (0.88-0.98) | 0.85 | 0.85 | 0.85 | 0.81 | 0.88 |
| Validation phase 1 | 0.62 | 0.92 (0.85-0.99) | 0.81 | 0.84 | 0.82 | 0.81 | 0.84 |
| Validation phase 2 | 0.62 | 0.98 (0.96-1.00) | 0.88 | 1.00 | 0.93 | 0.85 | 1.00 |
| Mild vs. Moderate |  |  |  |  |  |  |  |
| Discovery phase |  |  |  |  |  |  |  |
| Discovery set 1 | 0.57 | 0.95 (0.87-1.00) | 0.84 | 1.00 | 0.89 | 0.75 | 1.00 |
| Discovery set 2 | 0.57 | 0.99 (0.97-1.00) | 0.92 | 1.00 | 0.95 | 0.88 | 1.00 |
| Validation phase 1 | 0.57 | 0.64 (0.42-0.85) | 0.65 | 0.63 | 0.64 | 0.45 | 0.79 |
| Validation phase 2 | 0.57 | 1.00 (0.99-1.00) | 0.96 | 1.00 | 0.98 | 0.92 | 1.00 |
| Mild vs. Severe |  |  |  |  |  |  |  |
| Discovery phase |  |  |  |  |  |  |  |
| Discovery set 1 | 0.71 | 0.97 (0.94-1.00) | 0.91 | 1.00 | 0.93 | 0.69 | 1.00 |
| Discovery set 2 | 0.71 | 0.95 (0.9-1.00) | 0.88 | 0.93 | 0.90 | 0.70 | 0.98 |
| Validation phase 1 | 0.71 | 0.92 (0.83-1.00) | 0.88 | 0.75 | 0.85 | 0.67 | 0.92 |
| Validation phase 2 | 0.71 | 0.94 (0.91-0.98) | 0.82 | 0.97 | 0.94 | 0.75 | 1.00 |
| Moderate vs. Severe |  |  |  |  |  |  |  |
| Discovery phase |  |  |  |  |  |  |  |
| Discovery set 1 | 0.66 | 0.75 (0.61-0.8) | 0.64 | 0.74 | 0.67 | 0.45 | 0.86 |
| Discovery set 2 | 0.66 | 0.93 (0.89-0.98) | 0.85 | 0.85 | 0.85 | 0.73 | 0.92 |
| Validation phase 1 | 0.66 | 0.97 (0.93-1.00) | 0.88 | 0.94 | 0.91 | 0.84 | 0.96 |
| Validation phase 2 | 0.66 | 0.97 (0.95-1.00) | 0.90 | 0.93 | 0.91 | 0.81 | 0.96 |

**The results of ROC curve**
