# Supplementary material for: Metabolomics identifies and validates serum androstenedione as novel biomarker for diagnosing primary angle closure glaucoma and predicting the visual field progression
Source: eLife. 2024 Feb 15;12:RP91407. doi: 10.7554/eLife.91407 (PMC10942597; doi:10.7554/eLife.91407)
Supplement: Supplementary file 9. [file elife-91407-supp9.docx]

**Supplementary file 9**

|  | No progression (n=53) | Progression (n=44) | t value | P value |
| --- | --- | --- | --- | --- |
| Age (year) | 64.78±9.58 | 57.66±14.04 | 2.97 | 0.022 |
| Gender (male/female) | 26/27 | 22/22 | 0.009 | 0.93 |
| Diabetes (yes/no) | 11/42 | 6/38 | 0.84 | 0.36 |
| Hypercholesterolemia (yes/no) | 4/49 | 3/41 | 0.02 | 1 |
| Hypertension (yes/no) | 20/33 | 11/33 | 1.79 | 0.18 |
| IOP (mm Hg) | 20.83±10.20 | 21.51±11.26 | 0.31 | 0.72 |
| VCDR | 0.69±0.21 | 0.62±0.25 | 1.50 | 0.036 |
| CCT (um) | 546.76±53.82 | 549.10±52.65 | 0.21 | 0.58 |
| ACD (mm) | 2.00±0.59 | 2.33±0.69 | 2.50 | 0.14 |
| AL (mm) | 22.73±1.34 | 23.76±1.91 | 3.08 | 0.052 |
| MD (dB) | 9.29±5.87 | 11.44±6.65 | 1.70 | 0.093 |
| Androstenedione (ng/ml) | 1.59±2.11 | 2.12±0.72 | 4.10 | <0.001 |

**Comparison of Characteristics of No Progression and Progression Group in PACG Patients**
