# Supplementary material for: Metabolomics identifies and validates serum androstenedione as novel biomarker for diagnosing primary angle closure glaucoma and predicting the visual field progression
Source: eLife. 2024 Feb 15;12:RP91407. doi: 10.7554/eLife.91407 (PMC10942597; doi:10.7554/eLife.91407)
Supplement: Supplementary file 10. [file elife-91407-supp10.docx]

**Supplementary file 10**

|  | Difference in AUC | P value |
| --- | --- | --- |
| Discovery set |  |  |
| Androstenedione vs DHA | 0.255718085 | 2.99824E-09 |
| Androstenedione vs FFA(18:4) | 0.21356383 | 7.06683E-08 |
| Androstenedione vs FFA(22:6) | 0.255718085 | 2.99824E-09 |
| Validation set 1 |  |  |
| Androstenedione vs DHA | 0.002625 | 0.947989359 |
| Androstenedione vs FFA(18:4) | -0.1505 | 1.08683E-07 |
| Androstenedione vs FFA(22:6) | 0.002625 | 0.947989359 |

**Comparison of AUCs value among DHA, FFA (22:6), FFA (18:4), and androstenedione**
